# Convergent Hybrid Ablation and Concomitant Left Atrial Appendage Exclusion for Stroke Prevention and Rhythm Control in Persistent Atrial Fibrillation

**DOI:** 10.3390/jcm15093440

**Published:** 2026-04-30

**Authors:** Yonas R. Toma, Sune Damgaard, Christian L. Carranza

**Affiliations:** Department of Cardiothoracic Surgery, Rigshospitalet, University of Copenhagen, Blegdamsvej 9, 2100 Copenhagen, Denmark

**Keywords:** atrial fibrillation, stroke prevention, left atrial appendage exclusion, convergent procedure, hybrid ablation, rhythm control

## Abstract

**Background/Objectives:** Persistent and long-standing persistent atrial fibrillation (AF) presents a therapeutic clinical challenge balancing complex rhythm management with a heightened stroke risk. The left atrial appendage (LAA) is the primary source of thromboembolisms in these patients. This study evaluated the safety and efficacy of combining LAA exclusion with Convergent Hybrid Ablation for stroke prevention and rhythm control in a refractory patient cohort. **Methods**: A single-center observational cohort study was conducted including 28 patients with symptomatic persistent or long-standing persistent AF. The cohort was highly refractory, with 82.1% having failed at least one endocardial catheter ablation. The hybrid procedure consisted of sub-xiphoid epicardial ablation, thoracoscopic LAA exclusion (AtriClip), and endocardial catheter ablation. Safety and efficacy were assessed at 3 months and 12 months. **Results**: LAA exclusion was successfully performed in 96.4% of patients. The peri-operative safety profile was acceptable, with zero procedure-related strokes or deaths. At the 12-month follow-up, the rate of stroke or any other major adverse events was at 0.0%. Freedom from AF was 75.0%, shown by a 12-lead electrocardiography (ECG). Freedom from any atrial arrhythmia off anti-arrhythmic drugs (AADs) was achieved in 50.0% of patients. A total of 32.1% of the cohort required catheter ablation within 12 months to maintain sinus rhythm as part of the hybrid treatment. **Conclusions**: Concomitant LAA exclusion during Convergent Hybrid Ablation is a safe procedure with a high clinical success rate in maintaining sinus rhythm in a highly complex AF patient group. While no thromboembolic events were observed at 12 months, larger studies with longer follow-up are needed to confirm the potential for long-term stroke risk reduction. The findings suggest that for many patients, the hybrid procedure should be viewed as part of a multi-step strategy often requiring endocardial “touch-up” ablation.

## 1. Introduction

Atrial fibrillation (AF) is the most common cardiac arrhythmia in clinical practice, posing a significant threat to patient quality of life by increasing, among other things, the risk of stroke, heart failure, and all-cause mortality [1,2,3,4]. Among the most feared complications of AF is thromboembolic stroke [1,5]. Because the atria quiver chaotically, blood flow can become stagnant, especially in the left atrial appendage (LAA) [6,7,8]. This anatomical structure is where up to 90% of all AF-related blood clots form [6,9]. If a clot breaks free and travels to the brain, it causes a stroke, which is often more severe and disabling than strokes from other sources [1,7]. Consequently, effective stroke prevention is the primary therapeutic goal in the management of these patients.

When AF occurs, the pathophysiology of the disease begins to change [1,10]. Paroxysmal AF can result in a self-perpetuating process, changing the structure of the atrial tissue and making the electrical conductivity a complex maze [6,10]. This stems from fibrosis and inflammation among other things, which alter the posterior wall of the left atrium to an independent source of arrhythmia [6,10,11].

The most used intervention is catheter ablation, specifically complete isolation of the pulmonary veins (PVI) [1,11,12,13]. PVI is very effective (80% success rate) in paroxysmal AF patients but has only shown moderate effects in persisting AF due to its more complex nature [10,11,14,15]. The failure to maintain sinus rhythm leaves refractory patients at a continuously heightened risk of thromboembolic events, requiring alternative treatment strategies [13,15]. Surgical interventions are more relevant in these cases.

Historically, the surgical “Cox–Maze” procedure has been effective for persistent AF by creating a pattern of epicardial scar tissue to eliminate erratic electrical signals [4,6,12]. However, the traditional Maze procedure is highly invasive, requiring open-heart surgery, and is therefore nearly solely used in patients undergoing a concomitant cardiac operation [6,16].

To address the limitations of conventional catheter ablation and the surgical “Cox–Maze” procedure, the Convergent Hybrid Ablation procedure was developed [11,14,16]. It was developed to be a minimally invasive two-stage procedure that combines epicardial surgical ablation and endocardial catheter ablation [11,14,16]. The first stage involves a cardiac surgeon ablating the posterior wall of the left atrium epicardially through a sub-xiphoid incision. During this stage, the procedure is often supplemented with a thoracoscopic approach to epicardially occlude the LAA. While percutaneous endocardial LAA occlusion devices (e.g., WATCHMAN (Boston Scientific, Marlborough, MA, USA) or Amplatzer Amulet (Abbott, Abbott Park, IL, USA)) are widely utilized and have recently shown evidence for stroke prevention [17], epicardial exclusion using a device like the AtriClip PRO (AtriCure, Inc., Mason, OH, USA) offers the advantage of complete mechanical isolation without leaving foreign material inside the heart [6,7,16,18,19]. The second stage of the hybrid procedure is conventional PVI performed by an electrophysiologist that ablates the gaps remaining from the first procedure. The combination of two procedures ensures a more comprehensive treatment for persisting and long-standing persistent AF, which is a condition that lacks effective treatment [11,14,16]. By combining rhythm control with mechanical LAA exclusion, this dual approach directly targets both the arrhythmia and the primary source of stroke.

Clinical data regarding the long-term efficacy and safety of this dual procedure remain limited, particularly in highly refractory patients who have previously failed multiple endocardial catheter ablations. Understanding the clinical impact of combining LAA exclusion with hybrid ablation is essential for optimizing treatment in this complex patient group.

The aim of this study is to evaluate the clinical outcomes, with a specific focus on stroke prevention via concomitant LAA exclusion, alongside the safety and efficacy of the Convergent Hybrid Ablation procedure for the treatment of persistent AF in a single-center cohort of 28 patients.

## 2. Materials and Methods

### 2.1. Study Design and Patient Population

This study is a single-center, observational cohort study conducted at The Heart Centre, Rigshospitalet, Denmark. The study included 28 patients with symptomatic persistent or long-standing persistent atrial fibrillation (AF) who underwent a Convergent Hybrid Ablation procedure combined with LAA exclusion.

Between October 2022 and December 2024, consecutive patients presenting with symptomatic persistent or long-standing persistent AF were evaluated for the hybrid approach. The primary inclusion criteria required patients to be highly symptomatic (EHRA class ≥ 2) and to have previously failed at least one endocardial catheter ablation. While a formal screening log of all referred and rejected patients was not prospectively maintained, all patients meeting these criteria who consented to the procedure were included in the cohort.

Absolute exclusion criteria included previous cardiac surgery, unstable coronary artery disease, acute myocardial infarction or stroke within 90 days, inability to tolerate anticoagulation, Barrett’s esophagus, or a known left atrial thrombus. Relative exclusion criteria included, among others, a left ventricular ejection fraction (LVEF) <30%, NYHA Class III heart failure, severe pulmonary hypertension, and significant chronic kidney disease (CKD III or higher). A full list of the inclusion and exclusion criteria is available in the institutional protocol.

### 2.2. Pre-Operative Preparation

Patients were admitted pre-operatively (Day 0). Standard pre-operative evaluation included a review of medication status, pausing direct oral anticoagulants (DOACs) 1–2 days prior, routine blood work, and a 12-lead electrocardiography (ECG). Existing imaging, including echocardiography and cardiac CT or MRI, was reviewed by the operating surgeon. Prophylactic proton-pump inhibitors (Pantoprazole 40 mg once daily) were initiated and prescribed for 6 weeks to protect the esophagus.

### 2.3. Surgical and Electrophysiological Procedure (Hybrid Approach and LAA Exclusion)

The surgical procedure was performed as a two-stage operation within a single anesthetic session, intentionally combining epicardial ablation with mechanical stroke prevention by thoracoscopic LAA closure. Through subxiphoid surgical access to the pericardium, a scope and ablation equipment (EPi-Sense^®^ (AtriCure, Inc., Mason, OH, USA)) were inserted. Using a radiofrequency ablation catheter with a suction cup, accompanied by a 12-lead esophageal temperature probe (S-CATH™, CIRCA Scientific, Inc., Englewood, CO, USA) and pericardial coolant, the posterior wall of the left atrium and pulmonary veins were epicardially ablated. Crucially for stroke prevention, this procedure was supplemented with a left-sided thoracoscopic pericardial opening to access and safely occlude the left atrial appendage (LAA) using an AtriClip PRO^®^ device (AtriCure, Inc., Mason, OH, USA). The clip was applied under direct transesophageal echocardiography (TEE) guidance. Intraoperatively, if the residual LAA stump was measured to be greater than 5 mm, the clip was re-applied or relocated to ensure optimal exclusion.

### 2.4. Post-Operative Management and Follow-Up

Post-operatively, patients were transferred to the intensive care unit for observation before being moved to the surgical ward later the same day. Pleural drains were typically removed within 24 h. Post-operative chest X-ray was performed on day 2. The first 10 patients underwent echocardiography to check for pericardial effusion as an initial safety precaution, but it was deemed unnecessary for the following patients. Patients were discharged on post-operative day 3 or 4 following a final ECG.

All patients were scheduled for an outpatient clinical visit 3 months post procedure. At 12 months post procedure, a telephone consultation and a review of the electronic patient record, including a clinical assessment and an ECG, were performed. All data were prospectively collected and registered in a RedCap database.

### 2.5. Study Endpoints

The study endpoints were defined as follows, to evaluate both rhythm control and stroke prevention:

Stroke and Safety Endpoints: The primary safety endpoint was the incidence of peri-operative and post-operative major adverse events, focusing on the occurrence of stroke, transient ischemic attack (TIA), or procedure-related mortality at the 12-month follow-up.

Efficacy Endpoints: The primary efficacy endpoint was defined as freedom from any atrial arrhythmia without the use of any anti-arrhythmic drugs (AADs) assessed at 12 months. Secondary endpoints included freedom from any atrial arrhythmia regardless of the use of AADs assessed at 12 months.

### 2.6. Statistical Analyses

Descriptive statistics were used to summarize baseline characteristics, procedural details, and clinical outcomes. Continuous variables are presented as the mean ± standard deviation (SD) for normally distributed data, or as the median with interquartile range (IQR) for non-normally distributed data. Categorical variables are expressed as absolute frequencies (*n*) and relative percentages (%). For follow-up data with missing values, percentages were calculated based on the total number of patients with available data for that specific endpoint. To account for the small sample size, 95% confidence intervals (CIs) for categorical outcomes and success rates were calculated using the exact binomial (Clopper-Pearson) method. Data management and initial capture were performed using RedCap (version 16.0.15).

## 3. Results

A total of 28 patients underwent a two-stage Convergent Hybrid Ablation procedure including LAA exclusion. The following data were extracted: pre-operative data, peri-operative data, post-operative data, 3-month follow-up data and 12-month follow-up data from electronic patient journals. A total of 4 patients were lost to 12-month follow-up. One of the patients died by unknown reasons not related to the procedure, and the other three patients were unreachable and considered lost to follow-up.

### 3.1. Patient Characterictics

The mean age was 65.1 ± 8.9 years, and the majority were male (85.7%). This cohort represented a highly refractory patient group; 82.1% (*n* = 23) had at least one previously failed catheter ablation and the group as a whole had a mean of 2.4 ± 1.5 prior ablation procedures. Furthermore, 53.6% (*n* = 15) were diagnosed with long-standing persistent AF. The patients had a mean EHRA class of 2.6 ± 0.7 (*n* = 27) (moderate symptoms with patient troubled by symptoms/severe symptoms with normal daily activity affected). The mean left ventricular ejection fraction (LVEF) was 49.9% ± 12.7% and the mean Body Mass Index (BMI) was 28.6 ± 4.9 kg/m^2^. Hypertension was present in 51.9% (*n* = 14) of the cohort. Baseline characteristics are summarized in Table 1.

### 3.2. Procedural Outcomes

**Figure 1:**
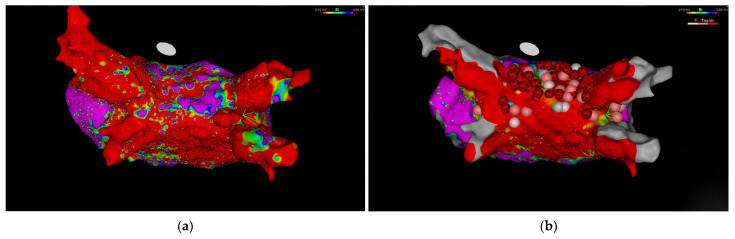
Representative left atrial voltage maps (posterior view) from a patient undergoing the second stage of the Convergent procedure. (**a**) A baseline endocardial voltage map obtained prior to the catheter touch-up, illustrating the epicardial posterior wall ablation created during the surgical stage. (**b**) The final voltage map after endocardial catheter ablation, demonstrating closure of conduction gaps and complete isolation of the posterior wall and pulmonary veins. Red/gray areas indicate low voltage (scar tissue), while purple indicates healthy myocardium.

The two-stage hybrid procedure was performed successfully in all 28 patients. Crucially for the stroke prevention strategy, concomitant left atrial appendage (LAA) exclusion via an epicardial AtriClip was successfully performed in 96.4% (*n* = 27) of the cohort. Acutely, 100% (*n* = 28) of patients were confirmed to be in sinus rhythm upon completion of the procedure (end of operation ECG) which reflects acute procedural restoration. Procedural time was recorded for a subgroup of 11 patients, showing a mean duration of 187.5 ± 24.0 min. The extensive epicardial posterior wall ablation and the subsequent endocardial completion of the box lesion are illustrated in Figure 1. Procedural outcomes are summarized in Table 2.

### 3.3. Safety Profile

The median total length of hospital stay was 3 days (IQR: 3–4), with no patients requiring prolonged admission in the intensive care unit. The overall in-hospital complication rate was 25.0% (*n* = 7), consisting primarily of single, non-fatal events, including infections (7.1%), symptomatic pleural effusion (3.6%), pneumothorax (3.6%), transient acute kidney injury (3.6%), pneumonia (3.6%), and vasovagal syncope (3.6%).

Importantly, regarding the primary safety endpoints, there were zero procedure-related strokes or deaths. At the 12-month follow-up, the incidence of stroke or transient ischemic attack (TIA) remained at 0.0% (Table 3).

### 3.4. Efficacy and Rhythm Outcomes

During the early post-operative phase, recurrence of atrial fibrillation or atrial flutter (AFL) was common, detected on ward ECGs in 50.0% (*n* = 14) of patients. This early recurrence led to an in-hospital Direct Current (DC) conversion in 28.6% (*n* = 8) of the cohort. Readmissions within the 3-month follow-up period occurred in 39.3% (*n* = 11) of patients. The primary reason for readmission was atrial arrhythmia requiring DC conversion, which was performed in 32.1% (*n* = 9) of the patients. Despite these early arrhythmic events, clinical improvement was evident by the 3-month follow-up. At this stage, 85.7% (*n* = 24) of patients were free from any atrial arrhythmia, documented by ECG. 66.7% (*n* = 18) of patients were entirely asymptomatic (EHRA 1), and 88.9% (*n* = 24) reported only mild or no symptoms (EHRA 1 or 2).

At the 12-month follow-up, 75.0% (*n* = 18) of patients were free from AF, and 62.5% (*n* = 15) were free from any atrial arrhythmia (AF/AFL). The primary efficacy endpoint, freedom from any atrial arrhythmia off anti-arrhythmic drugs, was achieved in 50.0% (*n* = 10) of patients (*N* = 20). 57.1% (*n* = 12) of the patients remained asymptomatic (EHRA 1) at 12 months. When comparing baseline symptomatic burden to the 12-month follow-up for patients with complete records (*n* = 21), 15 patients (71.4%) experienced a clinically meaningful reduction of at least one EHRA class. However, to maintain sinus rhythm over the 12-month period, 32.1% (*n* = 9) of the total cohort required repeated endocardial catheter ablation as expected due to the hybrid concept (Table 4).

## 4. Discussion

### 4.1. Principal Findings

This study evaluated the clinical outcomes, safety, and efficacy of the Convergent Hybrid Ablation procedure in a highly refractory cohort of 28 patients with symptomatic persistent and long-standing persistent atrial fibrillation. The cohort was characterized by advanced disease, with 82.1% of patients having previously failed at least one endocardial catheter ablation, presenting with a mean of 2.4 prior ablation procedures.

The main finding is that the procedure resulted in 50.0% of patients being free from any atrial arrhythmia without the use of anti-arrhythmic drugs at the 12-month follow-up. While the procedure demonstrated a favorable safety profile regarding major adverse events, the rate of repeat interventions was notable, with 32.1% of patients requiring a repeat catheter ablation within the first year.

Regarding thromboembolic protection, the LAA exclusion via an epicardial AtriClip was successfully achieved in 96.4% of the patients. Consequently, at the 12-month follow-up, there were zero procedure-related deaths and a 0.0% incidence of stroke or TIA.

### 4.2. Rhythm Control in a Refractory Population

The primary endpoint of this study was freedom from arrhythmia off anti-arrhythmic drugs, achieved in 50.0% of patients, which is comparable to the results of the multicenter randomized CONVERGE trial, which reported a success rate of 53.5% off anti-arrhythmic drugs [11]. However, our efficacy rate appears lower than that reported in the “real-world” London study, which reported a 68.8% success rate off anti-arrhythmic drugs at 12 months [14]. This difference in results is likely explained by significant differences in baseline patient complexity. The cohort in this study represents an exceptionally refractory population with 82.1% of our patients who had undergone at least one previously failed catheter ablation and up to 6 prior ablations. In contrast, the London study reported a prior failed ablation rate of only 38.8% [14]. Furthermore, the CONVERGE trial excluded patients with a left atrial diameter >6.0 cm, whereas our real-world cohort included patients with advanced structural remodeling [11].

The 50% success rate in maintaining sinus rhythm off AADs is highly meaningful. It demonstrates that the epicardial posterior wall ablation provides an effective method for stabilizing the heart’s rhythm, particularly in this treatment-resistant patient population. This outcome perfectly aligns with the CONVERGE trial, which found that the comprehensive hybrid procedure delivers a more robust and durable solution than traditional catheter ablation [11,16].

Another notable finding in this study was the high rate of subsequent catheter interventions. At 12 months, 32.1% of the patients had undergone a new endocardial catheter ablation procedure. While this appears higher than the re-ablation rate of 16.9% reported in the London study [14] and 5.7% in the PRECEPT trial comparisons [20], it was an expected part of our clinical protocol. In our cohort, patients accepting the treatment offer were guaranteed an endocardial catheter laboratory intervention if they presented with atrial fibrillation and/or a recurrence of clinical symptoms at their 3-month visit.

This planned, proactive intervention strategy directly addresses the chronicity of atrial fibrillation in this specific cohort. Long-standing atrial fibrillation is associated with extensive atrial fibrosis, which frequently requires these endocardial touch-ups to close gaps in the epicardial lesion set or to treat post-procedural atrial flutters. In the London study, 30.8% of patients experienced atrial tachycardia or flutter post surgery, often requiring cardioversion or ablation [14]. Similarly, our data showed that while 75% of patients were free from atrial fibrillation, only 62.5% were free from any arrhythmia, indicating that atrial flutter is a common sequela of the hybrid approach [15].

### 4.3. Safety Profile and Stroke Prevention

The overall post-surgery complication rate in our study was 25.0%. While this figure appears higher than the 7.8% complication rate in the CONVERGE trial [11] and the 4.5% complication rate in the London study [14], this is largely due to differences in the definition of a complication. The CONVERGE and London studies focused primarily on major complications such as stroke and death. When filtering our data for similar severe adverse events (symptomatic pleural effusion and pneumothorax), our severe complication rate was 7.1% (2/28). This is consistent with the safety profile in the literature [4,21].

Importantly, we observed no atrioesophageal fistulas or procedure-related deaths. A vital aspect of the safety profile in this cohort was the absence of peri-operative or long-term thromboembolic events. Stroke remains one of the most feared and disabling complications of AF, and to lessen the risk of stroke, concomitant epicardial LAA exclusion using the AtriClip was successfully achieved in 96.4% of the cohort. Only one patient who did not receive LAA exclusion was due to a permanent pacemaker being situated across the surgical field. This aligns with conclusions from the recent literature. A systematic review by Toale et al. with 922 patients concluded that epicardial LAA exclusion using the AtriClip is highly effective, achieving a 97.8% occlusion rate without any device-related adverse events [18]. Furthermore, they reported an exceptionally low post-procedural stroke or TIA incidence ranging from 0.2 to 1.5 per 100 patient-years, emphasizing the value of concomitant LAA exclusion during the Convergent Hybrid procedure, offering thromboembolic protection alongside rhythm control [7,18,19,22,23].

### 4.4. Study Limitations

This study has several limitations that should be acknowledged. First, the single-center observational design and relatively small cohort size limits the generalizability of the findings to broader populations. Second, data collection for the 3- and 12-month follow-ups was conducted retrospectively, leading to missing data points for some variables (e.g., anti-arrhythmic drug status at 12 months was only available for 20 patients). Finally, the assessment of rhythm outcomes relied primarily on standard 12-lead ECGs obtained during outpatient follow-up visits. In other randomized trials, like the CONVERGE trial, continuous monitoring strategies could have been used, such as 24 h and 7-day Holter monitors [11]. This difference in monitoring suggests that the true arrhythmia burden in our cohort might be underestimated compared to the rigorous monitoring performed in the CONVERGE trial. Furthermore, while the procedure demonstrated a 0.0% stroke rate at 12 months, the study is markedly underpowered to detect significant differences in thromboembolic events given the small sample size and short follow-up duration. Thus, the absence of strokes should be interpreted primarily as an indicator of procedural safety and feasibility rather than definitive statistical evidence of long-term stroke prevention efficacy.

## 5. Conclusions

In a highly symptomatic patient population with advanced atrial fibrillation and a history of multiple failed ablations, the Convergent Hybrid Ablation procedure combined with left atrial appendage (LAA) exclusion demonstrated an excellent safety profile. Concomitant LAA exclusion was successfully achieved in 96.4% of cases, resulting in no strokes or transient ischemic attacks at the 12-month follow-up. Regarding rhythm control, the procedure demonstrated freedom from arrhythmia off anti-arrhythmic drugs of 50.0% at 12 months, even in this heavily burdened cohort that had largely exhausted conventional percutaneous treatment options.

While the procedure is safe, the high rate of touch-up catheter ablation of 32.1% confirms the complexity of treating this refractory cohort. These findings indicate that while surgical ablation effectively restores sinus rhythm in end-stage atrial fibrillation patients and offers thromboembolic protection, it should clinically be viewed as a part of a multi-step management strategy rather than a standalone intervention, no matter if the percutaneous ablation is performed before or after surgery.

## Figures and Tables

**Table 1:** Overview of patient demographics, atrial fibrillation history and baseline measurements.

Characteristic	Total	*n* (Total = 28)
Demographics and AF History		
Age (years), mean (SD)	65.1 (SD: 8.9)	28
Male, *n* (%)	24 (85.7%)	28
AF duration > 1 year (LSPAF), *n* (%)	15 (53.6%)	28
Prior ablations, mean (SD)	2.4 (SD: 1.5)	28
Previous failed ablation, *n* (%)	23 (82.1%)	28
Baseline Measurements		
Body mass index (kg/m^2^), mean (SD)	28.6 (SD: 4.9)	28
Body mass index > 30 kg/m^2^, *n* (%)	9 (32.1%)	28
LVEF (%), mean (SD)	49.9 (SD: 12.7)	28
LVEF < 40%, *n* (%)	4 (14.3%)	28
$\mathrm{CH}A_{2}DS_{2}\text{-}\mathrm{VASc}$ score, median (IQR)	2.5 (IQR: 1–3)	28
Baseline Symptom Scores		
EHRA class, mean (SD)	2.6 (SD: 0.7)	27
NYHA class, mean (SD)	1.8 (SD: 0.8)	23
CCS class, mean (SD)	1.3 (SD: 0.8)	22
Comorbidities		
Hypertension, *n* (%)	14 (51.9%)	27
Diabetes, *n* (%)	1 (3.6%)	28
Hypercholesterolemia, *n* (%)	16 (57.1%)	28
Prior stroke/TIA, *n* (%)	3 (10.7%)	28
COPD, *n* (%)	1 (3.6%)	28
Kidney disease, *n* (%)	4 (14.3%)	28

Abbreviations: SD: Standard Deviation; LSPAF: Long-Standing Persistent Atrial Fibrillation; LVEF: Left Ventricular Ejection Fraction; EHRA: European Heart Rhythm Association; NYHA: New York Heart Association; CCS: Canadian Cardiovascular Society; TIA: Transient Ischemic Attack; COPD: Chronic Obstructive Pulmonary Disease.

**Table 2:** Summary of procedural details and acute outcomes for Convergent Hybrid Ablation procedure.

Characteristic	Total	*n* (Total = 28)
Procedural Details		
Total procedure time (min), mean (SD)	187.5 (SD: 24.0)	11
Blood loss (mL), mean (SD)	3.6 (SD: 18.9)	28
LAA exclusion (AtriClip), *n* (%)	27 (96.4%, 95% CI: 81.7–99.9%)	28
Acute Procedural Outcome		
Immediate sinus rhythm at exit, *n* (%)	28 (100.0%, 95% CI: 87.7–100.0%)	28

**Table 3:** Safety profile, peri-operative complications, and thromboembolic events.

Safety Outcome	Total	n (Total = 28)
Hospitalization & Survival		
Length of hospital stay (days), median (IQR)	3 (IQR: 3–4)	28
Intensive care admission, *n* (%)	0 (0.0%)	28
Procedure-related mortality, *n* (%)	0 (0.0%)	28
Thromboembolic Events (12 Months)		
Stroke, *n* (%)	0 (0.0%)	28
Transient Ischemic Attack (TIA), *n* (%)	0 (0.0%)	28
Post-operative Complications		
Any post-operative complications, *n* (%)	7 (25.0%, 95% CI: 10.7–44.9%)	28
Infection, *n* (%)	2 (7.1%)	28
Symptomatic pleural effusion, *n* (%)	1 (3.6%)	28
Pneumothorax, *n* (%)	1 (3.6%)	28
Transient acute kidney injury, *n* (%)	1 (3.6%)	28
Pneumonia, *n* (%)	1 (3.6%)	28
Vasovagal syncope, *n* (%)	1 (3.6%)	28

**Table 4:** Efficacy outcomes, symptom status, and re-intervention rates at 3- and 12-month follow-ups.

Outcome Parameter	3 Months	12 Months
Efficacy Outcomes		
Freedom from Atrial Fibrillation (ECG), *n*/*N* (%)	24/28 (85.7%, 95% CI: 67.3–96.0%)	18/24 (75.0%, 95% CI: 53.3–90.2%)
Freedom from any Atrial Arrhythmia (AF/AFL), *n*/*N* (%)	24/28 (85.7%, 95% CI: 67.3–96.0%)	15/24 (62.5%, 95% CI: 40.6–81.2%)
Freedom from AF off AADs, *n*/*N* (%)	-	10/20 (50.0%, 95% CI: 27.2–72.8%)
On Anti-Arrhythmic Drug (AAD) therapy, *n*/*N* (%)	-	10/20 (50.0%, 95% CI: 27.2–72.8%)
Symptom Status (EHRA Classification)		
Asymptomatic (EHRA 1), *n*/*N* (%)	18/27 (66.7%)	12/21 (57.1%)
Mild/Moderate symptoms (EHRA 2), *n*/*N* (%)	6/27 (22.2%)	3/21 (14.3%)
Severe symptoms (EHRA 3), *n*/*N* (%)	3/27 (11.1%)	5/21 (23.8%)
Disabling symptoms (EHRA 4), *n*/*N* (%)	0/27 (0.0%)	1/21 (4.8%)
Safety & Re-intervention		
Readmission (any cause), *n*/*N* (%)	11/28 (39.3%)	14/23 (60.9%)
Readmission for AF/AFL *, *n*/*N* (%)	9/28 (32.1%)	13/28 (46.4%)
Repeat endocardial catheter ablation (RFA), *n*/*N* (%)	0/28 (0.0%)	9/28 (32.1%, 95% CI: 15.9–52.4%)

* At the 3-month follow-up, readmissions for AF/AFL were specifically noted as requiring DC conversion. Note: Denominators (*N*) vary due to missing data points at follow-up (e.g., mortality, loss to follow-up, or incomplete electronic records).

## Data Availability

Data available on request.

## Abbreviations

The following abbreviations are used in this manuscript:

AAD	Anti-Arrhythmic Drug
AF	Atrial Fibrillation
AFL	Atrial Flutter
BMI	Body-Mass Index
CCS	Canadian Cardiovascular Society
CKD	Chronic Kidney Disease
COPD	Chronic Obstructive Pulmonary Disease
DC	Direct Current
DOAC	Direct Oral Anticoagulant
ECG	Electrocardiography
EHRA	European Heart Rhythm Association
LAA	Left Atrial Appendage
LVEF	Left Ventricular Ejection Fraction
IQR	Interquartile Range
n	Frequency
NYHA	New York Heart Association
PVI	Pulmonary Vein Isolation
RFA	Radiofrequency Ablation
SD	Standard Deviation
TIA	Transient Ischemic Attack
